# Mesmerism and Dental Surgery

**Published:** 1849-07

**Authors:** 


### MESMERISM AND DENTAL SURGERY.

Mesmerism appears to have its advantages, but we certainly never hoped, in our wildest moments of *esprit de corps*, to see tooth-drawing a matter of mere amusement; and we have to compliment Mr. Prideaux (see Elliotson's numerous cases) on the original idea of inviting a *party* to see a young lady's decayed stumps extracted. But Mr. Prideaux is a man of genius, in his way; the manner in which he describes his patient as "sitting with her hands folded in her lap, her countenance placid and serene, and her whole attitude that of repose," is really quite poetical; while the lady's amusement, at finding herself toothless, is a dramatic touch of the first water.

To be serious, we have often observed, with wonder and regret, that some of the most respectable practitioners of Dental Surgery have a tendency to quackery—with regret that an art, yielding in scientific requisites to few, and in usefulness to none, should be injured in respectability by such a junction. To the common scum of the profession, men, who having failed in every thing else, have adopted dentism as a *dernier resort*, we do not allude; quackery is *their* element, as necessary to them as the air they breathe; vulgar and uneducated as men; clumsy and unscientific as practitioners; sordid in mind, and bankrupt in principle, caring for no earthly thing but gain—for the fee—the reward of their labors and their patient's suffering—without quackery they could not exist; but for their lying, unblushing advertisements, equally disgraceful to them and insulting to the public, their practice would soon fall into desuetude, and their pockets become as empty as their heads—these men would adopt mesmerism or any other *ism*, that would promote their drawing an extra stump, or enable them to poison a few more patients, by filling their mouths with copper plate and earthenware masticators. But that men, who have any claim to respectability, any character to lose, should adopt a practice so questionable, and indulge in such contemptible mummery, does, we must confess, move our wonder and regret.

Who Mr. Prideaux is, except as far as he has himself chosen to enlighten us, we know not; from his advertisement in Dr. Elliotson's book, we learn that he is a dentist at Southampton. That he is a respectable and good natured man we have no doubt, but has he any (professional) character to spare, that he has thought fit to mix himself up with an art existing only in the wild and heated imaginations of the enthusiastic and the visionary? Does he suppose that his patients will respect a judgment warped by the mesmeric mania? Can he not take warning by others, who have sacrificed professional station and worldly interest at its shrine; or is he, too, anxious to become a martyr? It may be all very well for a physician, whose private fortune renders him independent of practice, to set public opinion at defiance, and ride the expensive hobby of mesmerism; but it is a dangerous experiment for a man to make, who is dependent on his profession; and we doubt whether Mr. Prideaux's mesmeric practice will compensate him for the loss of that of a more legitimate kind.

That the adoption of mesmerism is an excellent method of advertising, we allow. Who, for instance, ever heard of Mr. Gardner the surgeon, and Mr. Martin the dentist, gentlemen who honor Portsmouth with their company, till they made mesmerism perform the office of master of the ceremonies, and introduce them to the public, through the medium of the *Hampshire Telegraph.* It is an excellent method, because, while the mesmerists hail them as brothers, those who have no faith in the gentle mystery, *may* impute the absence of pain to Mr. Martin's skill in deducting the tooth; though really we cannot see any necessity for his *prolonging the wrench.* We have always found tooth-drawing, under the most favorable circumstances, sufficiently painful; quite enough so to test the powers of endurance; and the idea of making the young lady luxuriate in the operation by *prolonging the wrench,* seems to us a piece of gratuitous brutality.

Suppose, only for one moment, that the trance or coma, or whatever name it is to be called, had *not* rendered the patient insensible to pain; suppose it *did not,* and that her bearing it was a mere exertion of fortitude, induced by a little mischievous waggery at the expense of the professors, we question whether she really thanked Mr. Martin for the prolonged enjoyment.

But, really, these mesmerist tooth-drawers take rather unwarrantable liberties with the persons of their patients; one gentleman coolly thrusts a needle into a patient's arm, to ascertain whether he is insensible to pain, and in a fit state to be operated upon; another applies a heated glass stopper to test the *sensibility* of temperature, and *prickings* with a common needle to test that of *ordinary sensation.* We wonder what test he has to ascertain the common sense of the operator? But Mr. Prideaux is a wholesale mesmeric tooth-drawer, and we suppose requires some regular test to ascertain when his patient is fit for the wrench.*

Mr. Carstairs, however, a practitioner at Sheffield, must certainly be of Irish extraction, or by some accident lets out the truth as to the success of his operations. After informing the doctor (Elliotson) that his experiments have not been so numerous or important *per se*, he goes on to state, that in two cases in which he has extracted teeth, "*neither of the patients was aware, on being aroused from the state of mesmeric coma, that the teeth had been extracted*," (we quote the doctor's own words)—we dare say not; and if every gentleman would make as candid a confession, mesmeric coma would soon be rated at its true value.

But the surgeons seem determined that the dental practitioners shall not monopolize all the ridiculous notoriety of mesmerism. A Doctor Engledue seems to be a perfect Sancho in the service; not only catering for his dentist friends, but, now and then, doing a little on his own account. Verily, the Doctor must be a treasure to the old maiden ladies at Southsea; not only furnishing accounts of his own exploits, but raking up all the mesmeric intelligence from the colonies.

---

*We remember witnessing an exhibition at the London Mechanics' Institution, where an eminent Dentist thrust a sharp pointed instrument into the infra-orbital foramen, merely for amusement.

Mesmerism seems to be becoming the fashion, and in times like these, when so many want employment, we dare say the operators will have little difficulty in finding *operatees*, provided they are well paid, and the experiments are not *too painful*. Now we think if some of the mesmerisers—the gentlemen who are so fond of corking pins and hot sealing wax, would themselves submit to the ordeal, it would do more to establish the truth of the art, than all their experiments on others. If Mr. Martin, for instance, will let us take out a tooth for him with a *prolonged wrench*, or Dr. Elliotson take the place of the Okeys and let us make a pincushion of his back—let them be in the most satisfactory state of mesmeric coma—and if *they* bear these trifling inflictions without flinching, we will venture to say that they will secure a host of converts.

We remember, some years ago, a surgeon at Richmond, who, after the discovery of a slight *faux pas*, that went to prove the organ of amativeness, sent out cards of invitation to tea and Gospel. This was strangely anomalous, but we shall soon, no doubt, have invitations to coffee and operations, with corking pins and hot sealing wax, and a little tooth-drawing, or the amputation of a limb as a divertisement—the party to eat and drink by mesmeric sympathy.—*London Forceps.*

